# Altered Brain Functional Hubs and Connectivity in Type 2 Diabetes Mellitus Patients: A Resting-State fMRI Study

**DOI:** 10.3389/fnagi.2018.00055

**Published:** 2018-03-06

**Authors:** Daihong Liu, Shanshan Duan, Chaoyang Zhou, Ping Wei, Lihua Chen, Xuntao Yin, Jiuquan Zhang, Jian Wang

**Affiliations:** ^1^Department of Radiology, Southwest Hospital, Third Military Medical University (Army Medical University), Chongqing, China; ^2^Department of Endocrinology, The Third Affiliation Hospital of Chongqing Medical University, Chongqing, China; ^3^Department of Endocrinology, Southwest Hospital, Third Military Medical University (Army Medical University), Chongqing, China

**Keywords:** type 2 diabetes mellitus, resting-state functional MRI, degree centrality, Granger causality analysis, functional connectivity, cognitive impairment

## Abstract

Type 2 diabetes mellitus (T2DM) affects a vast population and is closely associated with cognitive impairment. However, the mechanisms of cognitive impairment in T2DM patients have not been unraveled. Research on the basic units (nodes or hubs and edges) of the brain functional network on the basis of neuroimaging may advance our understanding of the network change pattern in T2DM patients. This study investigated the change patterns of brain functional hubs using degree centrality (DC) analysis and the connectivity among these hubs using functional connectivity and Granger causality analysis. Compared to healthy controls, the DC values were higher in the left anterior cingulate gyrus (ACG) and lower in the bilateral lateral occipital cortices (LOC) and right precentral gyrus (PreCG) in T2DM patients. The functional connectivity between the left ACG and the right PreCG was stronger in T2DM patients, whereas the functional connectivity among the right PreCG and bilateral LOC was weaker. A negative causal effect from the left ACG to left LOC and a positive effect from the left ACG to right LOC were observed in T2DM patients, while in healthy controls, the opposite occurred. Additionally, the reserve of normal brain function in T2DM patients was negatively associated with the elevated glycemic parameters. This study demonstrates that there are brain functional hubs and connectivity alterations that may reflect the aberrant information communication in the brain of T2DM patients. The findings may advance our understanding of the mechanisms of T2DM-related cognitive impairment.

## Introduction

Type 2 diabetes mellitus (T2DM) affects 415 million individuals and is predicted to increase to 642 million in 2040, according to the Diabetes Atlas 7th Edition published by the International Diabetes Federation (http://www.idf.org/about-diabetes/facts-figures). Numerous studies have suggested that T2DM is closely associated with cognitive impairment, including the domains of motor function, executive function, processing speed and memory (Palta et al., 2014). Clarification of the underlying mechanism of cognitive impairment in T2DM patients for diagnosis and therapeutic effect estimation is essential before these patients develop dementia.

As a proven informative neuroimaging method, functional magnetic resonance imaging (fMRI) has been extensively applied to investigate alterations of brain function in T2DM patients. In fMRI studies, T2DM patients manifest functional changes in certain brain regions, and these changes are different from those associated with normal aging. For instance, the abnormal amplitude of low-frequency fluctuation, regional homogeneity, and functional connectivity (FC) in T2DM patients have been associated with poor performance in cognitive tests (Xia et al., 2013; Chen et al., 2014; Cui et al., 2014, 2015; Moheet et al., 2015). These studies have focused on local spontaneous brain activity or have analyzed the FC or network within the selected brain regions based on a priori assumption. According to the graph theory, a network is defined as a set of pairwise relationships between the elements of a system, which formally consists of a set of edges that link a set of nodes (Barabasi and Oltvai, 2004; Petersen and Sporns, 2015). Network analysis offers a new conceptual framework to investigate the network biology of aging (Wolfson et al., 2009; Tacutu et al., 2011), T2DM (Sandor et al., 2017) and neurodegenerative diseases (de Haan et al., 2017) at a variety of levels of scale including genes, proteins, synapses, neurons, neuronal circuits, neuronal populations, and systems (Petersen and Sporns, 2015). Therefore, the aforementioned fMRI findings provide a clue to explore T2DM-related brain dysfunction from the perspective of macroscopic nodes and connectivity at the whole brain level, which may share universal laws of network.

Degree centrality (DC), a measure based on graph theory, provides an approach for identifying the candidate functional hubs in the diabetic brain. DC is defined as the number of links that are strongly correlated to a given voxel or node for a binary graph and enables whole brain analysis at the voxel level, which may avoid the bias caused by selecting brain regions according to a priori assumption (Buckner et al., 2009; Zuo et al., 2012). It also considers the weights of these links for a weighted graph, and the weighted version of DC is more robust against confounding factors (Zuo et al., 2012). Thus, it can quantify the importance of a node to the rest of the brain, and nodes with high DC are defined as hubs (Zuo et al., 2012). Therefore, DC enables an investigation of the complexity and patterns of the brain functional connectome in diseases, including the subsequent analysis of FC between the hubs and the rest of the brain (Cui et al., 2016), or the interactions among these functional hubs in the present study.

Connectivity depicts the relationships among functionally segregated brain systems and can be classified into undirected and directed functional connectivity. Traditional FC is often used to investigate the statistical dependencies among several given seed regions in an undirected manner (Biswal et al., 1995). Directed FC is explicitly used to explore the functional interactions in a directed manner and can be estimated using the Granger causality model (Seth et al., 2015). The positive casual effect and negative causal effect can be quantified with positive and negative signed-path coefficients and interpreted as activation and inhibition, respectively (Hamilton et al., 2011). Both undirected and directed FC are the components of functional integration that are used to analyze functional brain architectures (Friston et al., 2013). By combining the traditional FC and Granger causality analysis (GCA) approaches, more information can be obtained from different perspectives to map the connectivity pattern among the brain functional hubs that are probably affected by T2DM.

In this study, we hypothesized that the aberrant brain function of hubs and their connectivity may contribute to brain dysfunction in T2DM patients. The DC method was applied to identify the candidate functional hubs. Next, the FC and GCA methods were applied to investigate the connectivity among these functional hubs. We also investigated the relationships of brain functional alterations with clinical data and neuropsychological performance. Our study provides evidence to further understand the neurological mechanisms that underlie T2DM-associated cognitive impairment.

## Materials and methods

### Subjects

T2DM patients were recruited from inpatients and the community and healthy controls (HC) were recruited from the community between December 2013 and December 2015. Forty-seven T2DM patients and 47 healthy controls, who were matched with regard to age, sex, education and body mass index (BMI), were enrolled in the study. Both the T2DM patients and the healthy controls met the following inclusion criteria: (1) between 45 and 70 years old; 2) at least six years of education; and (3) right-handedness. T2DM was diagnosed by endocrinologists according to the criteria published by the World Health Organization in 1999 (Alberti and Zimmet, 1998). The T2DM patients had at least 1 year of disease duration. The exclusion criteria for all participants were as follows: (1) organic disease in the brain, such as stroke, tumor, or a white matter lesion rating score ≥ 2 (Wahlund et al., 2001); (2) physical disability; (3) pregnancy, thyroid dysfunction; (4) signs of dementia (Mini-Mental State Examination [MMSE] score ≤ 24) (Galea and Woodward, 2005), major depression (Hamilton Depression Rating Scale-24 item [HAMD] > 20) (Hamilton, 1960), or other psychiatric disorders; (5) severe hearing or visual impairment; and (6) contraindications to MRI. Patients with T2DM-related complications were also excluded, including diabetic foot, retinopathy, and nephropathy.

The study protocol was approved by the Medical Research Ethics Committee of the Southwest Hospital (Chongqing, China). Written informed consent was obtained from participants after they were informed of the study details. Additionally, the study process strictly obeyed the protocol.

### Clinical data

The following information about the subjects was recorded using a standardized protocol: handedness, height, weight, BMI [weight in kg]/[height in m]^2^), resting arm arterial blood pressure, medical history and current medications. For T2DM patients, we also recorded the date of diagnosis to calculate the disease duration. Venous blood samples were collected by venipuncture after overnight fasting for the biometric measurements, including glucose-related parameters (fasting plasma glucose, fasting insulin, fasting C-peptide, and glycosylated hemoglobin [HbA_1c_]), lipoid parameters (total cholesterol, triglyceride, high-density lipoprotein [HDL] cholesterol, low-density lipoprotein [LDL] cholesterol), renal function parameters (blood urea nitrogen, serum creatine, uric acid, and cystatin C), thyroid function parameters (free triiodothyronine [FT3], free thyroxine [FT4] and thyroid-stimulating hormone [TSH]), and homocysteine. The updated homeostasis model assessment of the insulin resistance (HOMA2-IR) index was calculated using the HOMA2 Calculator v2.2.3 software (http://www.dtu.ox.ac.uk/homacalculator/) to evaluate insulin resistance in all subjects.

### Neuropsychological tests

All participants underwent cognitive status assessments with a battery of neuropsychological tests in a fixed order that assessed their global cognitive level and major cognitive subdomains. The global level of cognition was evaluated with the MMSE and Montreal Cognitive Assessment (MoCA) tests. The depressive state was evaluated with HAMD to exclude cases with major depression. The major cognitive subdomains were evaluated with the following tests: (1) the Trail Making Test (TMT, parts A and B) for executive function and psychomotor speed (Bowie and Harvey, 2006); (2) the Verbal Fluency Test (VFT) for mental flexibility (Diamond, 2013); (3) the Digit Span Test (DST, forwards and backwards) for working memory (Diamond, 2013); and (4) the Auditory Verbal Learning Test (AVLT, including immediate recall, short-term delayed recall, long-term delayed recall, long-term delayed recognition and total score) for episodic memory (Zhao et al., 2015). A trained neuropsychologist administered the battery of tests and was blinded to the group status. Each participant completed the assessment in approximately 60 min.

### MRI data acquisition

MRI scan was performed on the same day that the clinical data were obtained and the cognitive test was performed. MRI data were acquired using a 3.0-T MR scanner (Trio, Siemens Medical, Erlangen, Germany) and a 12-channel head coil. Subjects were instructed to close their eyes, stay awake and avoid thinking about any topics. Earplugs and cushions were used to alleviate noise influence and restrict head motion, respectively. T2-weighted images: repetition time = 6,000 ms, echo time = 89 ms, flip angle = 120°, field of view = 230 × 230 mm^2^, slices = 20, thickness = 5.0 mm, matrix = 448 × 448 and voxel size = 0.5 × 0.5 × 5.0 mm^3^. Fluid-attenuated inversion recovery (FLAIR) images: repetition time = 9,000 ms, echo time = 93 ms, flip angle = 130°, field of view = 220 × 220 mm^2^, slices = 25, thickness = 4.0 mm, matrix = 256 × 256 and voxel size = 0.9 × 0.9 × 4.0 mm^3^. Resting-state functional images were collected using an echo planar imaging (EPI) sequence: repetition time = 2,000 ms, echo time = 30 ms, flip angle = 90°, field of view = 192 × 192 mm^2^, slices = 36, thickness = 3 mm, matrix = 64 × 64 and voxel size = 3 × 3 × 3 mm^3^; 240 volumes were transversely acquired. T1-weighted structural images were collected using a volumetric 3D magnetization prepared by rapid-acquisition gradient-echo (MP-RAGE) sequence: repetition time = 1,900 ms, echo time = 2.52 ms, flip angle = 9°, field of view = 256 × 256 mm^2^, slices = 176, thickness = 1 mm, matrix = 256 × 256 and voxel size = 1 × 1 × 1 mm^3^, sagittally scanned.

### MRI data analysis

No subjects were excluded after the T2-weighted and FLAIR images were reviewed by two radiologists with at least five years of work experience.

Structural and functional data analyses were performed using toolkits based on Statistical Parametric Mapping 8 software (SPM 8, http://www.fil.ion.ucl.ac.uk/spm). Intracranial tissue segmentation was performed with Voxel Based Morphometry Toolbox 8 software (VBM8, version 435) according to a previously described protocol (Whitwell, 2009). The main steps include spatial normalization to match every subject's T1-weighed images to the template image, and segmentation of the intracranial tissue into gray matter, white matter and cerebrospinal fluid, which automatically produces information about the volumes of each part of the brain tissue. Gray matter was smoothed with 4 mm full-width half-maximum.

Functional data were preprocessed using Data Processing Assistant for Resting-State fMRI (DPARSF module v4.1 of Data Processing & Analysis of Brain Imaging v2.1, http://rfmri.org/dpabi) according to the standard procedure (Yan and Zang, 2010). All DICOM files were converted to NifTI files. Next, the first 10 volumes were removed to enable the subjects to adapt to the scanning environment, especially the noise. Slice-timing was performed to correct the time differences between slices. Realignment was performed to correct head motion, and a report of head motion was created. Any subjects with head motion > 2.0 mm in any direction of x, y, and x or > 2.0° at any angle were excluded from the subsequent statistical analyses. Friston 24-parameter model was applied to regress out head motion effects (Yan et al., 2013). Other nuisance variables including white matter signal and cerebrospinal fluid signal were regressed out. Individual functional images were normalized into the Montreal Neurological Institute (MNI) space for intersubject comparison. The resulting images were smoothed with 4 mm full-width half-maximum. Detrending was applied to remove the systematic drift of the baseline signal. The data were bandpass filtered (0.01–0.08 Hz) to reduce physiological noise at other bands of frequency.

Based on preprocessing, DC calculations were performed using DPARSF in a voxel-wise manner with a threshold *r* > 0.25 in accordance with previous studies (Beucke et al., 2013; Mueller et al., 2013). Peak MNI coordinates of the candidate brain functional hubs were obtained via group comparison of DC maps and considered to be the center of the spherical region of interest (ROI) with a 6-mm radius. Connectivity among the ROIs was analyzed using rs-fMRI data analysis toolkits (REST v1.8, http://www.restfmri.net) in a ROI-wise manner, including FC and GCA analyses. For FC, the correlation coefficients between the ROIs were computed and normalized with Fisher's *r*-to-*z* transformation. For GCA, signed-path coefficients between ROIs were computed in a multivariate mode for the subsequent parametric statistical analyses (Hamilton et al., 2011).

### Statistical analysis

Inter-group comparisons of numeric data were conducted using SPSS software (version 20.0; IBM Corp., Armonk, NY) and included demographic data, clinical parameters, and neuropsychological test scores. First, the data distribution was verified using the Kolmogorov-Smirnov test. Second, independent samples of the *t*-test and Mann-Whitney U test were applied to normally distributed continuous data and to non-normally distributed data, respectively. The sex proportion was examined with the χ^2^ test. *p* < 0.05 indicated statistical significance.

Intra- and inter-group analyses of DC maps were conducted using REST software. A one-sample *t*-test was applied to investigate the DC pattern with the base of “0” in the intra-group. An independent *t*-test was applied to investigate the DC differences between the groups with age, sex, education, BMI, blood pressure, blood biometric parameters (except fasting glucose, fasting insulin, fasting C-peptide and HbA_1c_) entered as covariates. Gray matter maps were also included as covariates to control the influence of structural changes in the T2DM patients. The resulting maps were corrected for multiple comparisons using AlphaSim (*p* < 0.001, cluster size > 20 voxels, number of Monte Carlo simulations = 10,000, cluster connection radius: rmm = 5.0).

For the z scores of FC and signed-path coefficients, statistical analyses were performed using SPSS software. A one-sample *t*-test was applied to investigate the patterns of FC and GCA in each group. An independent sample *t*-test was applied to investigate the differences between T2DM patients and healthy controls in terms of FC and GCA. The relationships of DC and connectivity with clinical parameters and neuropsychological test scores were explored through partial correlation analyses in SPSS software within the T2DM group. Both the independent sample *t*-test and the correlation analysis were adjusted with gray matter volumes, and the same covariates that employed in the inter-group analyses of the DC maps were applied to control their possible effect on the results. The independent sample *t*-test was also controlled for multiple comparisons with Bonferroni correction (*p* < 0.05/6 for FC and *p* < 0.05/12 for GCA).

## Results

### Demographic and clinical data comparison

No significant inter-group differences were found in terms of age, sex, education, BMI, blood pressure, total cholesterol, LDL cholesterol, blood urea nitrogen, cystatin C, uric acid and TSH. The T2DM patients had significantly higher levels of glucose-related parameters, including fasting plasma glucose, fasting insulin, HbA_1c_ and HOMA2-IR index (all *p* < 0.05). The T2DM patients had significantly higher levels of triglycerides and homocysteine but lower fasting C-peptide and serum creatine levels (all *p* < 0.05). The inter-group differences in FT3 and FT4 were significant (all *p* < 0.05); however, the levels were within the normal range. The details are presented in Table 1.

**Table 1:** Demographic and clinical data of the subjects.

	**T2DM**	**HC**	***p-*value**
Age (years)	58.66 ± 6.87	57.36 ± 5.42	0.312
Sex (male:female)	28/19	25/22	0.533^a^
Education (years)	10.60 ± 3.06	10.77 ± 2.65	0.773
T2DM duration (years)	8.87 ± 6.61	–	–
BMI (kg/m^2^)	25.46 ± 5.11	23.94 ± 3.87	0.109
Gray matter (cm^3^)	608.15 ± 58.00	615.16 ± 51.67	0.538
White matter (cm^3^)	534.91 ± 64.85	524.16 ± 66.56	0.430
Brain parenchyma (cm^3^)	1143.06 ± 114.78	1139.32 ± 107.80	0.871
Systolic blood pressure (mmHg)	128.91 ± 16.81	134.55 ± 17.88	0.119
Diastolic blood pressure (mmHg)	79.49 ± 10.01	80.26 ± 10.25	0.715
HbA_1c_ (%)	8.26 ± 2.08	5.63 ± 0.39	<0.001
HbA_1c_ (mmol/mol)	66.76 ± 22.80	38.15 ± 4.33	<0.001
Fasting plasma glucose (mmol/L)	7.47 ± 2.70	5.22 ± 0.46	<0.001
Fasting insulin (mIU/L)	14.91 (9.58, 24.54)	11.41 (8.39, 17.10)	0.035^b^
Fasting C-peptide (ng/mL)	1.81 ± 1.05	2.22 ± 0.90	0.047
HOMA2-IR	0.28 (0.19, 0.52)	0.21 (0.16, 0.32)	0.015^b^
Total cholesterol (mmol/L)	4.89 ± 1.04	5.10 ± 1.02	0.325
Triglyceride (mmol/L)	1.45 (1.05, 1.98)	1.23 (0.91, 1.48)	0.023^b^
HDL cholesterol (mmol/L)	1.06 ± 0.23	1.41 ± 0.36	<0.001
LDL cholesterol (mmol/L)	3.16 ± 0.83	3.27 ± 0.76	0.489
Homocysteine (μmol/L)	16.62 ± 10.03	10.70 ± 4.10	<0.001
Blood urea nitrogen (mmol/L)	6.36 ± 2.45	5.72 ± 1.23	0.113
Serum creatine (μmol/L)	65.00 (57.00, 78.00)	78.00 (66.00, 85.00)	0.007^b^
Cystatin C (mg/L)	0.72 (0.64, 0.91)	0.76 (0.69, 0.86)	0.639^b^
Uric acid (μmol/L)	316.67 ± 79.72	328.34 ± 69.09	0.450
Free triiodothyronine, FT3 (pmol/L)	4.31 ± 0.91	5.13 ± 0.72	<0.001
Free thyroxine, FT4 (pmol/L)	15.52 ± 2.34	16.48 ± 1.89	0.032
Thyroid-stimulating hormone, TSH (mIU/L)	2.12 ± 0.95	2.57 ± 1.54	0.090

*p < 0.05 indicates statistical significance*.

a *χ^2^ test for sex (n)*.

b *Mann-Whitney U-test for non-normally distributed data [median (QR)]. Independent t-test for the other normally distributed continuous data (means ± SD). T2DM, type 2 diabetes mellitus; HC, healthy control; BMI, body mass index; HDL, high-density lipoprotein; LDL, low-density lipoprotein*.

### Neuropsychological test comparison

The T2DM patients exhibited poorer performance on the tests of MoCA, TMT-B, DST forwards and long-term delayed recall of AVLT (all *p* < 0.05). No significant inter-group differences were observed in the other tests. The details are presented in Table 2.

**Table 2:** Comparison of the neuropsychological test results between the two groups.

	**T2DM**	**HC**	***p*-value**
**GENERAL COGNITION**			
MMSE	29.00 (27.00, 29.00)	29.00 (28.00, 29.00)	0.203^a^
MoCA	23.17 ± 2.88	24.51 ± 2.31	0.015
**EXECUTIVE FUNCTION AND PSYCHOMOTOR SPEED**			
TMT-A	74.67 ± 41.11	64.17 ± 32.59	0.173
TMT-B	183.19 ± 89.90	142.00 ± 58.35	0.010
**MENTAL FLEXIBILITY**			
VFT	42.17 ± 7.33	40.55 ± 7.05	0.279
**WORKING MEMORY**			
DST forwards	8.83 ± 1.37	9.60 ± 1.50	0.011
DST backwards	4.00 (3.00, 5.00)	4.00 (3.00, 4.00)	0.911^a^
**EPISODIC MEMORY**			
AVLT immediate recall	6.46 ± 1.69	6.86 ± 1.49	0.226
AVLT short-term delayed recall	6.77 ± 3.18	7.74 ± 2.41	0.096
AVLT long-term delayed recall	5.40 ± 3.77	6.72 ± 2.43	0.047
AVLT long-term delayed recognition	11.00 (8.00, 13.00)	12.00 (9.00, 13.00)	0.327^a^
AVLT total score	28.97 ± 9.94	32.34 ± 7.45	0.065

*p < 0.05 was considered statistically significant*.

a *Mann-Whitney U-test for non-normally distributed data [median (QR)]. Independent t-test for the other normally distributed continuous data (means ± SD). MMSE, Mini-Mental State Examination; MoCA, Montreal Cognitive Assessment; TMT, Trail Making Test; VFT, Verbal Fluency Test; DST, Digital Span Test; AVLT, Auditory Verbal Learning Test*.

### Brain volume comparison

The general volumes of gray matter, white matter and brain parenchyma (the sum of gray and white matter) revealed no significant differences between the two groups (Table 1).

### DC analysis

**Figure 1:**
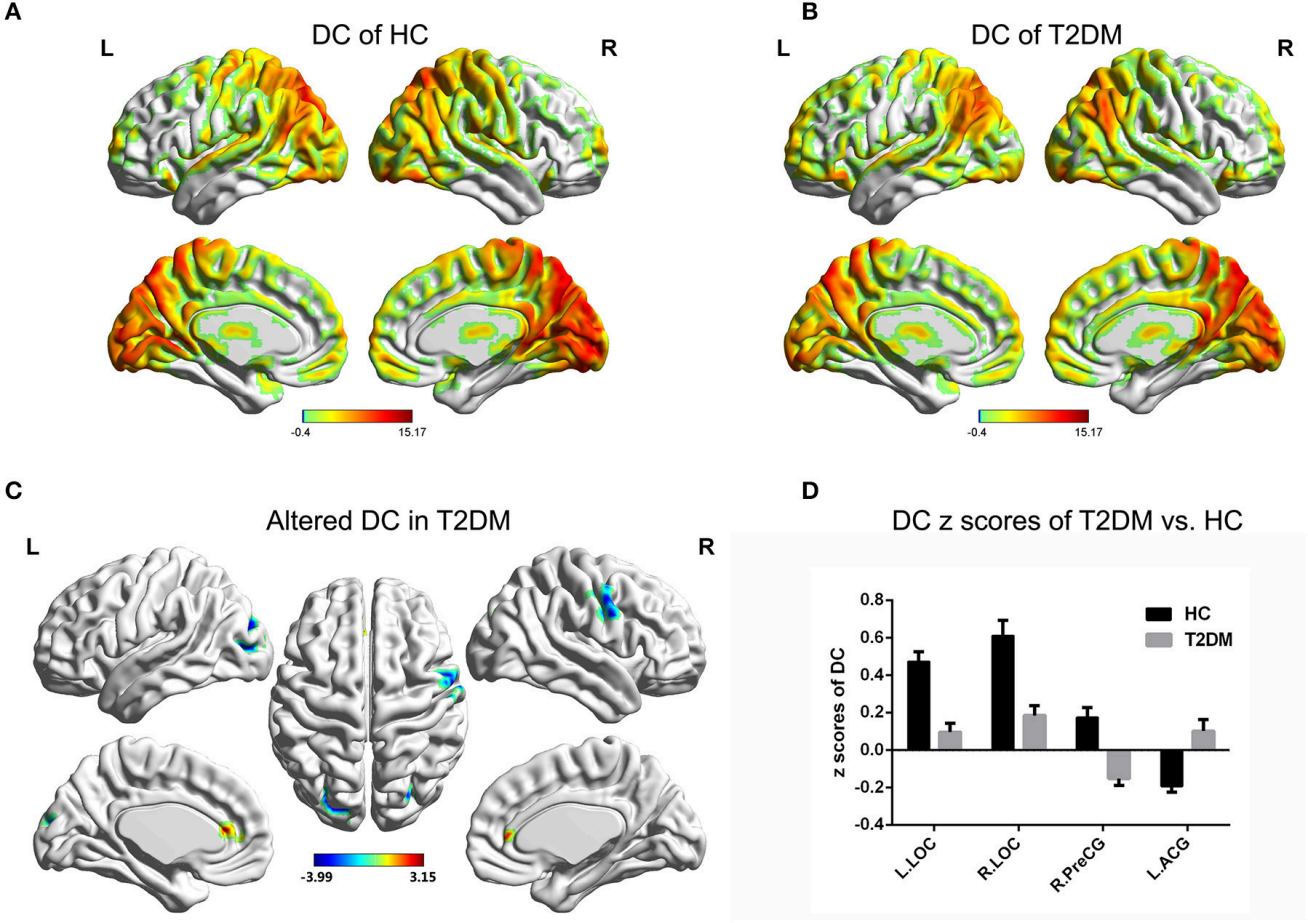
DC value distribution of intra-group and inter-group comparisons. **(A,B)** The spatial distribution of the DC value in the HC group and T2DM group. **(C)** The significantly altered DC map in the T2DM group. **(D)** Comparison of DC value between the two groups. AlphaSim corrected (*p* < 0.001, cluster size > 20 voxels). The color bar denotes the *t*-value. Error bars define the SEM. ACG, anterior cingulate gyrus; LOC, lateral occipital cortices; PreCG, precentral gyrus; R, right; L, left.

Compared to the global mean value, both groups displayed higher DC values in the posterior cingulate cortex, cuneus, angular gyrus, occipital cortex, superior frontal cortex, precentral gyrus and postcentral gyrus (Figures 1A,B). In the T2DM patients, significantly higher DC values were observed in the left anterior cingulate gyrus (ACG), and significantly lower DC values were observed in the bilateral lateral occipital cortices (LOC) and right precentral gyrus (PreCG). Details of the changed brain areas are presented in Table 3 (Figures 1C,D).

**Table 3:** Brain regions with significant DC differences between the two groups.

	**Brain regions**	**BA**	**Peak MNI**			***t*-value**	**Cluster (voxels)**
			***X***	***Y***	***Z***		
1	Left anterior cingulate gyrus	32	−9	42	9	3.1455	28
2	Right lateral occipital cortex	19	27	−78	24	−3.1327	20
3	Left lateral occipital cortex	19	−24	−84	21	−3.9907	147
4	Right precentral gyrus	43	63	−3	30	−3.4576	55

*MNI, Montreal Neurological Institute; BA, Broadmann Area. R, Right; L, left; AlphaSim corrected (p < 0.001, cluster > 20 voxels)*.

### Connectivity analysis

**Figure 2:**
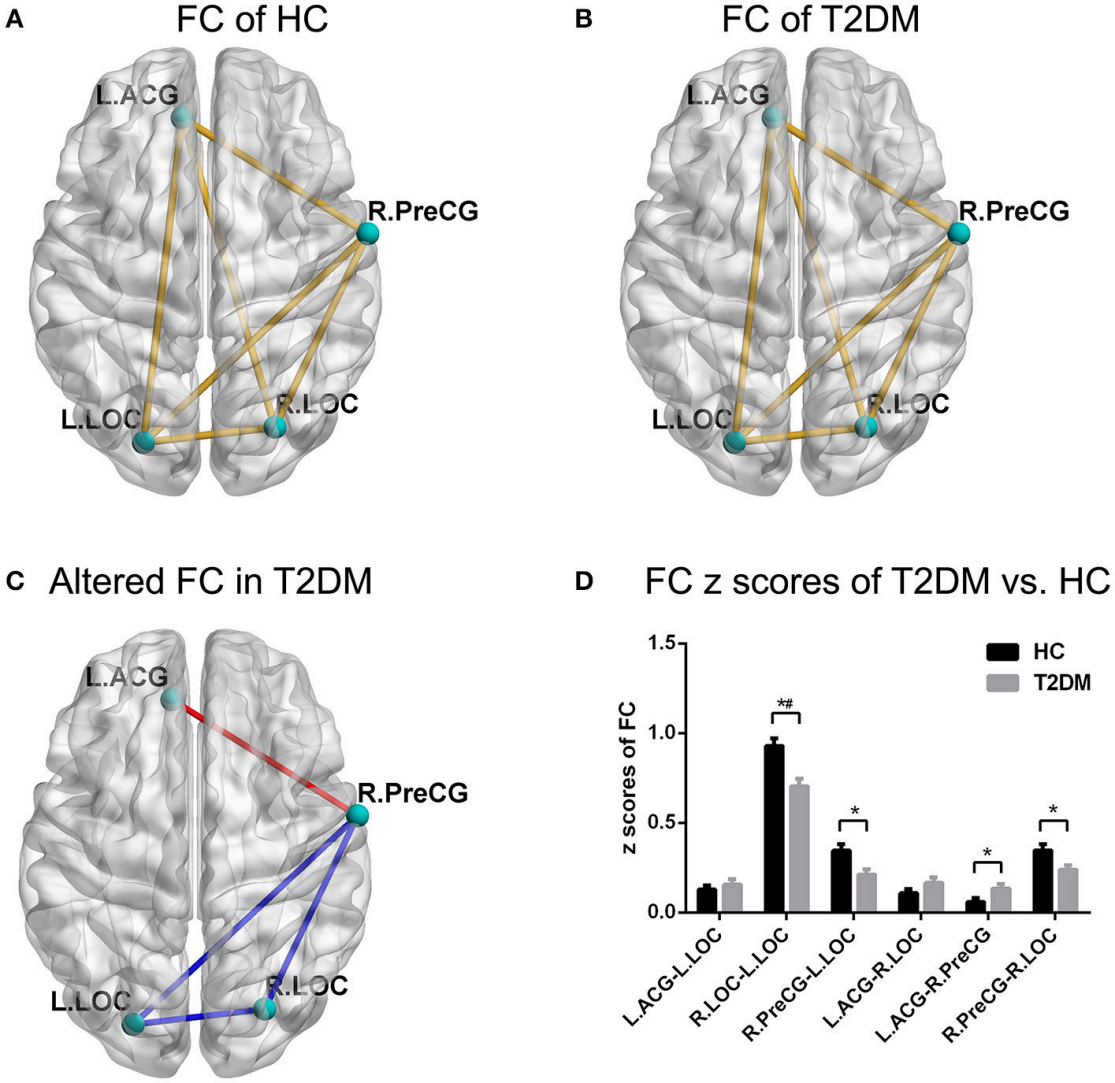
FC pattern of intra-group and inter-group comparisons. **(A,B**) The FC pattern in the HC group and T2DM group. **(C)** The significantly altered FC in the T2DM group. **(D)** Comparison of FC z scores between the two groups. ^*^p < 0.05, ^#^*p* < 0.05/6 (Bonferroni correction). Error bars define the SEM. ACG, anterior cingulate gyrus; LOC, lateral occipital cortices; PreCG, precentral gyrus; R, right; L, left.

For the FC analyses, the one-sample *t*-test suggested that FC existed in both groups among the ROIs, including the left ACG, right PreCG, and bilateral LOC (all *p* < 0.05; Figures 2A,B). The independent *t*-test suggested that the FC of the left ACG-right PreCG (*p* = 0.015) in T2DM patients was significantly stronger than that in the healthy controls, and the FC values of the left LOC-right PreCG (*p* = 0.011), right LOC-right PreCG (*p* = 0.019), and left LOC-right LOC (*p* = 0.001) were significantly weaker than those in the healthy controls (Figures 2C,D). After multiple comparisons correction, the FC of the left LOC-right LOC in T2DM patients was significantly weaker than that in the healthy controls (*p* < 0.05/6).

**Figure 3:**
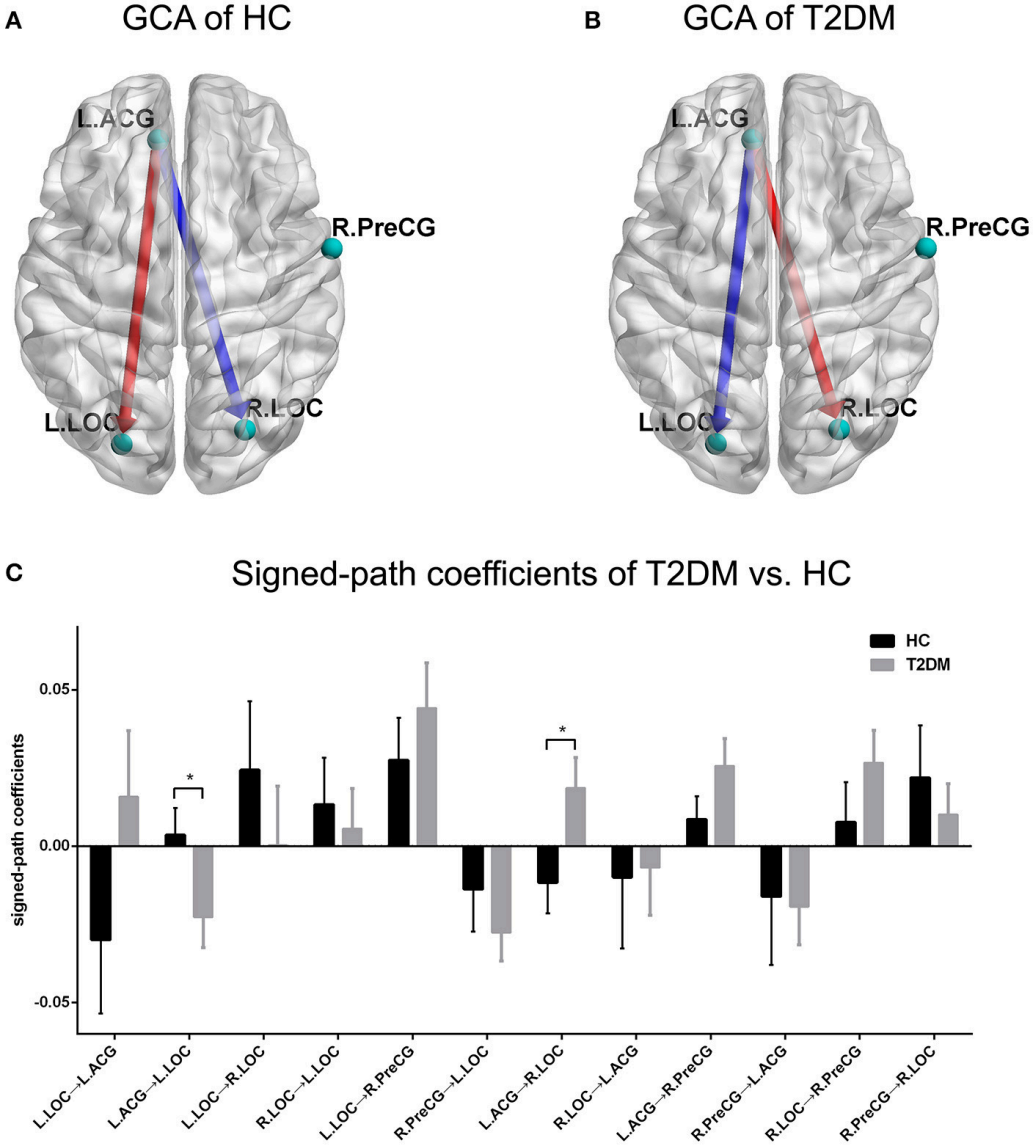
GCA pattern of intra-group and inter-group comparisons. **(A,B)** Causal effect patterns of the HC group and T2DM group. The red arrow indicates a positive casual effect and its direction. The blue arrow indicates a negative casual effect and its direction. **(C)** Comparison of signed-path coefficients between the two groups. ^*^p < 0.05. Error bars define the SEM.

With respect to the GCA analyses, the one-sample *t*-test showed a positive causal effect from the left ACG to the left LOC and a negative causal effect from the left ACG to the right LOC in the healthy controls (Figures 3A,C). However, the conditions were inverted such that a negative causal effect from the left ACG to the left LOC and a positive causal effect from the left ACG to the right LOC were observed in the T2DM patients (Figures 3B,C). Significant differences between the two groups in terms of the two directed functional edges were observed (*p* < 0.05; Figure 3C). Unfortunately, the independent *t*-test results of GCA could not bear the multiple comparisons correction (*p* > 0.05/12).

### Correlation analysis

**Figure 4:**
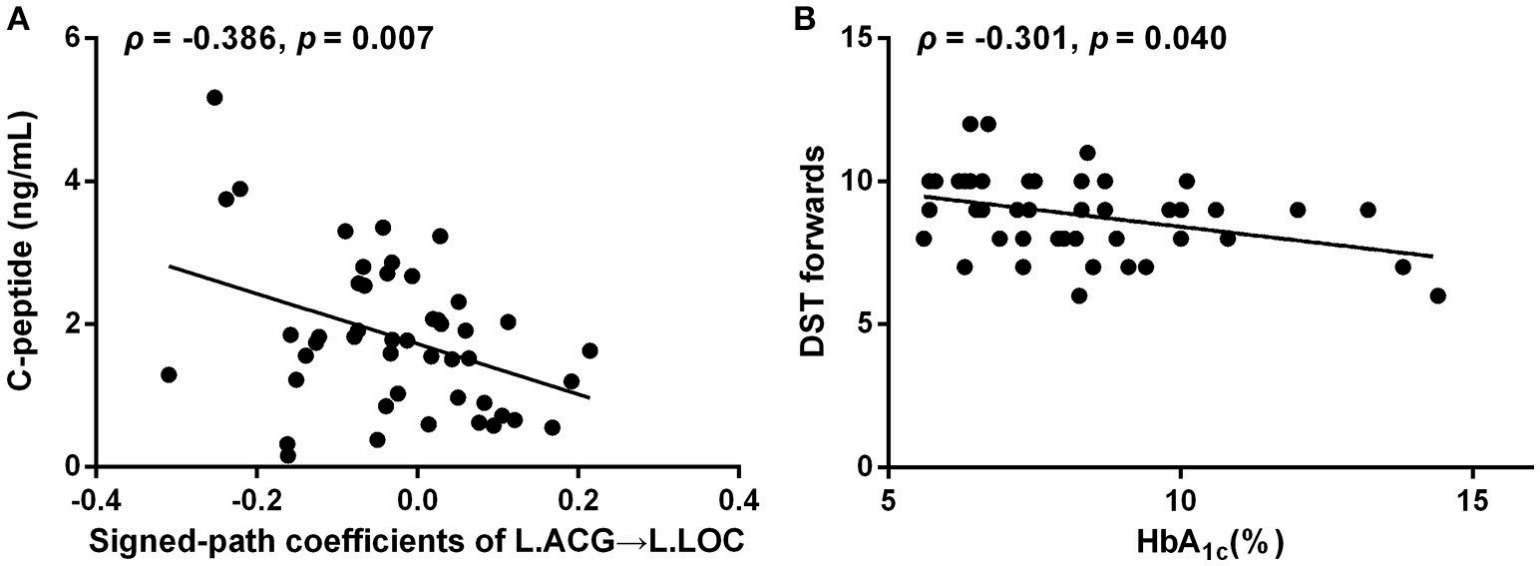
Correlations among the connectivity, neuropsychological performance and diabetes-related parameters. **(A)** Signed-path coefficients of the left ACG to the left LOC vs. C-peptide (ng/mL). **(B)** DST forwards vs. HbA_1c_(%).

Signed-path coefficients from the left ACG to the left LOC were negatively correlated with fasting C-peptide levels (ρ = −0.386, *p* = 0.007; Figure 4A) in T2DM patients. Poor performance on the DST forwards was associated with elevated HbA_1c_ levels in T2DM patients (HbA_1c_ [%], ρ = −0.301, *p* = 0.040; HbA_1c_ [mmol/mol], ρ = −0.301, *p* = 0.040; Figure 4B). However, no correlations were observed between disrupted DC or connectivity and lower scores on the neuropsychological tests or disease duration.

## Discussion

To explore the possible neurological mechanisms underlying cognitive dysfunction in T2DM patients, we combined the DC, FC and GCA approaches to investigate the changes in the candidate brain functional hubs and their connectivity. The increased DC in left ACG and decreased DC in the occipital areas were consistent with a previous study (Cui et al., 2016). A novel finding was the increased DC in the right PreCG. The changed FC pattern of right PreCG may be associated with impaired preparation and execution of goal-directed actions. The GCA approach further revealed the disordered direct connectivity from the left ACG to bilateral occipital areas.

The brain regions where identified hubs are located have been reported to be abnormal in previous studies using other neuroimaging metrics in T2DM patients, which suggests that these brain regions are susceptible to T2DM. The ACG is usually identified as the disturbed brain region across experiments and appeared with increased amplitude of low-frequency fluctuation, regional homogeneity (Cui et al., 2014; Liu D. et al., 2016), and cerebral blood flow (Cui et al., 2017). The aforementioned hyperactivity of ACG has usually been regarded as a means to compensate for the cognitive loss and maintain normal cognition (He et al., 2015; Liu D. et al., 2016; Cui et al., 2017). However, the mechanism of the compensation requires further research. In contrast, a decreased amplitude of low-frequency fluctuation and regional homogeneity were observed in the occipital lobe (Xia et al., 2013; Cui et al., 2014; Peng et al., 2016). Decreased connectivity of the PreCG was found with the posterior cingulate cortex (Chen et al., 2014) and the thalamic (Chen et al., 2015). These brain regions also exhibited gray matter atrophy in T2DM patients (Chen et al., 2012; Garcia-Casares et al., 2014; Peng et al., 2015). We discovered a change in the number of edges that connect the bilateral LOC and right PreCG, which rendered these brain regions candidate functional hubs to be damaged in T2DM patients (Zuo et al., 2012). These findings provided information about the reorganized layout of the brain network with a graph theory-based approach in addition to previous studies.

Placing the identified brain functional hubs into the context of brain local networks, the changed FC of the right PreCG may suggest impaired preparation and execution of goal-directed actions. The motor system participates in the constitution of internal representations of sensory information (Crowe et al., 2004) and obtains visually perceived information to prepare a motor response (Merchant and Georgopoulos, 2006). However, the decreased DC and FC values of the right PreCG indicated its decline in communication efficiency with the visual cortex and other brain regions. Additionally, the motor system is closely connected with the ACG which mediates focusing attention on targets to select appropriate actions (Isomura et al., 2003). The increased FC between the ACG and PreCG may be interpreted as brain efforts to guide motor behavior. Notably, the process of goal-directed action is a main constituent of executive function. Moreover, for its motor speed component, the poor executive performance in TMT-B may be the behavioral level evidence of cognitive and motor system dysfunction.

The GCA results may further suggest that the reorganization of the brain network in T2DM patients may be associated with the impaired visual information acquisition for executive function. The ACG serves an important role in the top-down control network (Petersen and Posner, 2012). The occipital cortex is an important brain area of vision-related information encoding for working memory which is the primary component of executive function (Bentley et al., 2004; Diamond, 2013). Furthermore, visual cortex can be mediated by the ACG which monitors and resolves conflict by enhancing the to-be-attended information and suppressing distracter stimuli (Petersen and Posner, 2012; Liu Y. et al., 2016). In this study, the disordered excitatory and inhibitory effects from the ACG to the visual cortex suggest that T2DM patients may experience a cognitive impairment in visual information acquisition that is indirectly related to the deficit in executive function. In addition, unlike the scale free organization of molecular networks (Budovsky et al., 2007; Tacutu et al., 2010), the human brain network was proposed to exhibit prominent small-world architecture which facilitates efficient information communication (Liao et al., 2017). The impacts of brain functional hubs and connectivity alterations on the small-world property remains to be further explored.

GCA connections appear at where FC connections exist; however, we observed that there was no overlap of significant alterations between undirected and directed connectivity in the present study. On one hand, undirected FC is usually assessed with the correlation coefficient, which can be regarded as descriptive (Biswal et al., 1995). On the other hand, the directed FC depicted by GCA rests explicitly on the linear vector autoregressive models (Seth et al., 2015). It is difficult to make a direct comparison between correlation data and a model. The non-overlapping alterations between the undirected and directed connectivity may result from the distinction of mathematical theories. It is also reported that internet gaming disorder individuals without FC alterations exhibited impaired GCA connections (Guller et al., 2012). This phenomenon requires a further investigation.

Our finding also suggested that the level of glycemic parameters may be linked to the reserve of normal brain function in T2DM patients. Elevated HbA_1c_ was negatively correlated with spontaneous brain activity in the middle temporal gyrus (Xia et al., 2013) and with cognitive performance such as VFT (Chen et al., 2014). The higher insulin resistance has been associated with decreased FC between the posterior cingulate cortex and middle temporal gyrus (Chen et al., 2014; Yang et al., 2016). With the higher elevation of C-peptide in this study, a greater deviation of directed FC was observed in patients than in the healthy controls. Additionally, the patients with lower HbA_1c_ levels obtained higher scores on the DST forwards. The results were similar to those of several previous studies, which indicated that T2DM patients with elevated glycemic parameters exhibited decreased/inverted brain functional activity and poor cognitive performance (Xia et al., 2013; Chen et al., 2014; Liu D. et al., 2016; Yang et al., 2016). However, the findings of this study did not provide sufficient information about the relationships between the altered brain function and cognitive performance. Additional studies are required to investigate these mysteries.

The main limitations of the present investigation are as follows: First, the study comprised a relatively small population. The inter-group comparisons of FC and GCA were unable to bear the multiple corrections. These factors may restrict its statistical power and the explanation of the results. Second, we deduced the visually related cognition impairment in T2DM patients according to fMRI metrics. However, the lack of assessment on this cognitive domain weakened the basis of our inference. Third, the approach of GCA is controversial due to the poor temporal resolution in fMRI studies. However, the GCA method remains a powerful tool to explore the directed connectivity and helps elucidate the complexity of the brain (Seth et al., 2015). A large-sample study with comprehensive cognitive assessments and the application of new technology, such as compressed sensing (Lustig et al., 2008), to accelerate the sampling may solve these problems in the future. We are also considering the application of other network measurements, for instance, betweenness, closeness, path length (Rubinov and Sporns, 2010), which could be combined with the molecular networks to map the complex biological systems of T2DM-related cognitive impairment in depth (Barabasi et al., 2011; Vidal et al., 2011).

## Conclusions

Our findings suggest that the DC of the left ACG, bilateral LOC and right PreCG, as well as the connectivity among them reflected by FC and GCA in different perspectives, are altered in T2DM patients. These alterations may be associated with the disruption of visual information acquisition and goal-directed action execution, both of which have previously proven to be related to executive function. Patients with lower glycemic parameters may reserve more normal brain functions. This study provides insight into the neurological underpinnings of T2DM-related cognitive impairment using neuroimaging.

## Author contributions

DL contributed to the experiments, data analysis and writing of the manuscript. SD contributed to performing the experiments and writing and revising the manuscript. CZ contributed to the data collection. PW designed the experiment and revised the manuscript. LC contributed to the data analysis and manuscript revision. XY contributed to the manuscript revision. JZ and JW are the guarantors of this study and had complete access to all data in the study. They accept responsibility for the integrity of the data and the accuracy of the data analysis.

### Conflict of interest statement

The authors declare that the research was conducted in the absence of any commercial or financial relationships that could be construed as a potential conflict of interest.

## Abbreviations

ACG: anterior cingulate gyrus
AVLT: Auditory Verbal Learning Test
BMI: body mass index
DC: degree centrality
DST: Digit Span Test
EPI: echo planar imaging
FC: functional connectivity
FLAIR: fluid-attenuated inversion recovery
fMRI: functional magnetic resonance imaging
FT3: free triiodothyronine
FT4: free thyroxine
GCA: Granger causality analysis
HAMD: Hamilton Depression Rating Scale
HbA_1c_: glycosylated hemoglobin
HC: healthy control
HDL: high-density lipoprotein
HOMA2-IR: updated homeostasis model assessment of insulin resistance
LDL: low-density lipoprotein
LOC: lateral occipital cortices
MMSE: Mini-Mental State Examination
MNI: Montreal Neurological Institute
MoCA: Montreal Cognitive Assessment
MP-RAGE: magnetization prepared by rapid-acquisition gradient-echo
PreCG: precentral gyrus
ROI: region of interest
T2DM: Type 2 diabetes mellitus
TMT: Trail Making Test
TSH: thyroid-stimulating hormone
VFT: Verbal Fluency Test.

## References

[B1] Alberti K. G. Zimmet P. Z. (1998). Definition, diagnosis and classification of diabetes mellitus and its complications. Part 1: diagnosis and classification of diabetes mellitus provisional report of a WHO consultation. Diabet. Med. 15, 539–553. 10.1002/(SICI)1096-9136(199807)15:7<539::AID-DIA668>3.0.CO;2-S 9686693

[B2] Barabasi A. L. Gulbahce N. Loscalzo J. (2011). Network medicine: a network-based approach to human disease. Nat. Rev. Genet. 12, 56–68. 10.1038/nrg2918 21164525 PMC3140052

[B3] Barabasi A. L. Oltvai Z. N. (2004). Network biology: understanding the cell's functional organization. Nat. Rev. Genet. 5, 101–113. 10.1038/nrg1272 14735121

[B4] Bentley P. Husain M. Dolan R. J. (2004). Effects of cholinergic enhancement on visual stimulation, spatial attention, and spatial working memory. Neuron 41, 969–982. 10.1016/S0896-6273(04)00145-X 15046728

[B5] Beucke J. C. Sepulcre J. Talukdar T. Linnman C. Zschenderlein K. Endrass T.. (2013). Abnormally high degree connectivity of the orbitofrontal cortex in obsessive-compulsive disorder. JAMA Psychiatry 70, 619–629. 10.1001/jamapsychiatry.2013.173 23740050

[B6] Biswal B. Yetkin F. Z. Haughton V. M. Hyde J. S. (1995). Functional connectivity in the motor cortex of resting human brain using echo-planar MRI. Magn. Reson. Med. 34, 537–541. 10.1002/mrm.1910340409 8524021

[B7] Bowie C. R. Harvey P. D. (2006). Administration and interpretation of the trail making test. Nat. Protoc. 1, 2277–2281. 10.1038/nprot.2006.390 17406468

[B8] Buckner R. L. Sepulcre J. Talukdar T. Krienen F. M. Liu H. Hedden T.. (2009). Cortical hubs revealed by intrinsic functional connectivity: mapping, assessment of stability, and relation to Alzheimer's disease. J. Neurosci. 29, 1860–1873. 10.1523/JNEUROSCI.5062-08.2009 19211893 PMC2750039

[B9] Budovsky A. Abramovich A. Cohen R. Chalifa-Caspi V. Fraifeld V. (2007). Longevity network: construction and implications. Mech. Ageing Dev. 128, 117–124. 10.1016/j.mad.2006.11.018 17116322

[B10] Chen Y. C. Jiao Y. Cui Y. Shang S. A. Ding J. Feng Y.. (2014). Aberrant brain functional connectivity related to insulin resistance in type 2 diabetes: a resting-state fMRI study. Diabetes Care 37, 1689–1696. 10.2337/dc13-2127 24658392

[B11] Chen Y. C. Xia W. Qian C. Ding J. Ju S. Teng G. J. (2015). Thalamic resting-state functional connectivity: disruption in patients with type 2 diabetes. Metab. Brain Dis. 30, 1227–1236. 10.1007/s11011-015-9700-2 26116166

[B12] Chen Z. Li L. Sun J. Ma L. (2012). Mapping the brain in type II diabetes: voxel-based morphometry using DARTEL. Eur. J. Radiol. 81, 1870–1876. 10.1016/j.ejrad.2011.04.025 21546180

[B13] Crowe D. A. Chafee M. V. Averbeck B. B. Georgopoulos A. P. (2004). Participation of primary motor cortical neurons in a distributed network during maze solution: representation of spatial parameters and time-course comparison with parietal area 7a. Exp. Brain Res. 158, 28–34. 10.1007/s00221-004-1876-3 15042265

[B14] Cui Y. Jiao Y. Chen H. J. Ding J. Luo B. Peng C. Y.. (2015). Aberrant functional connectivity of default-mode network in type 2 diabetes patients. Eur. Radiol. 25, 3238–3246. 10.1007/s00330-015-3746-8 25903712 PMC4595523

[B15] Cui Y. Jiao Y. Chen Y. C. Wang K. Gao B. Wen S.. (2014). Altered spontaneous brain activity in type 2 diabetes: a resting-state functional MRI study. Diabetes 63, 749–760. 10.2337/db13-0519 24353185

[B16] Cui Y. Li S. F. Gu H. Hu Y. Z. Liang X. Lu C. Q. (2016). Disrupted brain connectivity patterns in patients with type 2 diabetes. AJNR. Am. J. Neuroradiol. 37, 2115–2122. 10.3174/ajnr.A4858 PMC5201447 27365332

[B17] Cui Y. Liang X. Gu H. Hu Y. Zhao Z. Yang X. Y.. (2017). Cerebral perfusion alterations in type 2 diabetes and its relation to insulin resistance and cognitive dysfunction. Brain Imaging Behav. 11, 1248–1257. 10.1007/s11682-016-9583-9 27714551 PMC5653700

[B18] de Haan W. van Straaten E. C. W. Gouw A. A. Stam C. J. (2017). Altering neuronal excitability to preserve network connectivity in a computational model of Alzheimer's disease. PLoS Comput. Biol. 13:e1005707. 10.1371/journal.pcbi.1005707 28938009 PMC5627940

[B19] Diamond A. (2013). Executive functions. Annu. Rev. Psychol. 64, 135–168. 10.1146/annurev-psych-113011-143750 23020641 PMC4084861

[B20] Friston K. Moran R. Seth A. K. (2013). Analysing connectivity with Granger causality and dynamic causal modelling. Curr. Opin. Neurobiol. 23, 172–178. 10.1016/j.conb.2012.11.010 23265964 PMC3925802

[B21] Galea M. Woodward M. (2005). Mini-Mental State Examination (MMSE). Aust. J. Physiother. 51:198. 10.1016/S0004-9514(05)70034-9 16187459

[B22] Garcia-Casares N. Berthier M. L. Jorge R. E. Gonzalez-Alegre P. Gutierrez Cardo A. Rioja Villodres J.. (2014). Structural and functional brain changes in middle-aged type 2 diabetic patients: a cross-sectional study. J. Alzheimers. Dis. 40, 375–386. 10.3233/JAD-131736 24448784

[B23] Guller Y. Tononi G. Postle B. R. (2012). Conserved functional connectivity but impaired effective connectivity of thalamocortical circuitry in schizophrenia. Brain Connect. 2, 311–319. 10.1089/brain.2012.0100 23020103 PMC3621336

[B24] Hamilton J. P. Chen G. Thomason M. E. Schwartz M. E. Gotlib I. H. (2011). Investigating neural primacy in major depressive disorder: multivariate Granger causality analysis of resting-state fMRI time-series data. Mol. Psychiatry 16, 763–772. 10.1038/mp.2010.46 20479758 PMC2925061

[B25] Hamilton M. (1960). A rating scale for depression. J. Neurol. Neurosurg. Psychiatr. 23, 56–62. 10.1136/jnnp.23.1.56 14399272 PMC495331

[B26] He X. S. Wang Z. X. Zhu Y. Z. Wang N. Hu X. Zhang D. R.. (2015). Hyperactivation of working memory-related brain circuits in newly diagnosed middle-aged type 2 diabetics. Acta Diabetol. 52, 133–142. 10.1007/s00592-014-0618-7 24993663 PMC4416650

[B27] Isomura Y. Ito Y. Akazawa T. Nambu A. Takada M. (2003). Neural coding of “attention for action” and “response selection” in primate anterior cingulate cortex. J. Neurosci. 23, 8002–8012. 12954861 10.1523/JNEUROSCI.23-22-08002.2003 PMC6740492

[B28] Liao X. Vasilakos A. V. He Y. (2017). Small-world human brain networks: perspectives and challenges. Neurosci. Biobehav. Rev. 77, 286–300. 10.1016/j.neubiorev.2017.03.018 28389343

[B29] Liu D. Duan S. Zhang J. Zhou C. Liang M. Yin X.. (2016). Aberrant brain regional homogeneity and functional connectivity in middle-aged T2DM patients: a resting-state functional MRI study. Front. Hum. Neurosci. 10:490. 10.3389/fnhum.2016.00490 27729856 PMC5037166

[B30] Liu Y. Bengson J. Huang H. Mangun G. R. Ding M. (2016). Top-down modulation of neural activity in anticipatory visual attention: control mechanisms revealed by simultaneous EEG-fMRI. Cereb. Cortex 26, 517–529. 10.1093/cercor/bhu204 25205663 PMC4712792

[B31] Lustig M. Donoho D. L. Santos J. M. Pauly J. M. (2008). Compressed Sensing MRI. IEEE Signal Process. Mag. 25, 72–82. 10.1109/MSP.2007.914728

[B32] Merchant H. Georgopoulos A. P. (2006). Neurophysiology of perceptual and motor aspects of interception. J. Neurophysiol. 95, 1–13. 10.1152/jn.00422.2005 16339504

[B33] Moheet A. Mangia S. Seaquist E. R. (2015). Impact of diabetes on cognitive function and brain structure. Ann. N.Y. Acad. Sci. 1353, 60–71. 10.1111/nyas.12807 26132277 PMC4837888

[B34] Mueller S. Wang D. Fox M. D. Yeo B. T. Sepulcre J. Sabuncu M. R.. (2013). Individual variability in functional connectivity architecture of the human brain. Neuron 77, 586–595. 10.1016/j.neuron.2012.12.028 23395382 PMC3746075

[B35] Palta P. Schneider A. L. Biessels G. J. Touradji P. Hill-Briggs F. (2014). Magnitude of cognitive dysfunction in adults with type 2 diabetes: a meta-analysis of six cognitive domains and the most frequently reported neuropsychological tests within domains. J. Int. Neuropsychol. Soc. 20, 278–291. 10.1017/S1355617713001483 24555960 PMC4132660

[B36] Peng B. Chen Z. Ma L. Dai Y. (2015). Cerebral alterations of type 2 diabetes mellitus on MRI: a pilot study. Neurosci. Lett. 606, 100–105. 10.1016/j.neulet.2015.08.030 26306652

[B37] Peng J. Qu H. Peng J. Luo T. Y. Lv F. J. Chen L.. (2016). Abnormal spontaneous brain activity in type 2 diabetes with and without microangiopathy revealed by regional homogeneity. Eur. J. Radiol. 85, 607–615. 10.1016/j.ejrad.2015.12.024 26860674

[B38] Petersen S. E. Posner M. I. (2012). The attention system of the human brain: 20 years after. Annu. Rev. Neurosci. 35, 73–89. 10.1146/annurev-neuro-062111-150525 22524787 PMC3413263

[B39] Petersen S. E. Sporns O. (2015). Brain networks and cognitive architectures. Neuron 88, 207–219. 10.1016/j.neuron.2015.09.027 26447582 PMC4598639

[B40] Rubinov M. Sporns O. (2010). Complex network measures of brain connectivity: uses and interpretations. Neuroimage 52, 1059–1069. 10.1016/j.neuroimage.2009.10.003 19819337

[B41] Sandor C. Beer N. L. Webber C. (2017). Diverse type 2 diabetes genetic risk factors functionally converge in a phenotype-focused gene network. PLoS Comput. Biol. 13:e1005816. 10.1371/journal.pcbi.1005816 29059180 PMC5667928

[B42] Seth A. K. Barrett A. B. Barnett L. (2015). Granger causality analysis in neuroscience and neuroimaging. J. Neurosci. 35, 3293–3297. 10.1523/JNEUROSCI.4399-14.2015 25716830 PMC4339347

[B43] Tacutu R. Budovsky A. Fraifeld V. E. (2010). The NetAge database: a compendium of networks for longevity, age-related diseases and associated processes. Biogerontology 11, 513–522. 10.1007/s10522-010-9265-8 20186480

[B44] Tacutu R. Budovsky A. Yanai H. Fraifeld V. E. (2011). Molecular links between cellular senescence, longevity and age-related diseases - a systems biology perspective. Aging 3, 1178–1191. 10.18632/aging.100413 22184282 PMC3273898

[B45] Vidal M. Cusick M. E. Barabasi A. L. (2011). Interactome networks and human disease. Cell 144, 986–998. 10.1016/j.cell.2011.02.016 21414488 PMC3102045

[B46] Wahlund L. O. Barkhof F. Fazekas F. Bronge L. Augustin M. Sjogren M.. (2001). A new rating scale for age-related white matter changes applicable to MRI and CT. Stroke 32, 1318–1322. 10.1161/01.STR.32.6.1318 11387493

[B47] Whitwell J. L. (2009). Voxel-based morphometry: an automated technique for assessing structural changes in the brain. J. Neurosci. 29, 9661–9664. 10.1523/JNEUROSCI.2160-09.2009 19657018 PMC6666603

[B48] Wolfson M. Budovsky A. Tacutu R. Fraifeld V. (2009). The signaling hubs at the crossroad of longevity and age-related disease networks. Int. J. Biochem. Cell Biol. 41, 516–520. 10.1016/j.biocel.2008.08.026 18793745

[B49] Xia W. Wang S. Sun Z. Bai F. Zhou Y. Yang Y.. (2013). Altered baseline brain activity in type 2 diabetes: a resting-state fMRI study. Psychoneuroendocrinology 38, 2493–2501. 10.1016/j.psyneuen.2013.05.012 23786881

[B50] Yan C. G. Cheung B. Kelly C. Colcombe S. Craddock R. C. Di Martino A.. (2013). A comprehensive assessment of regional variation in the impact of head micromovements on functional connectomics. Neuroimage 76, 183–201. 10.1016/j.neuroimage.2013.03.004 23499792 PMC3896129

[B51] Yan C. G. Zang Y. F. (2010). DPARSF: a Matlab toolbox for “pipeline” data analysis of resting-state fMRI. Front. Syst. Neurosci. 4:13 10.3389/fnsys.2010.00013 20577591 PMC2889691

[B52] Yang S. Q. Xu Z. P. Xiong Y. Zhan Y. F. Guo L. Y. Zhang S.. (2016). Altered intranetwork and internetwork functional connectivity in type 2 diabetes mellitus with and without cognitive impairment. Sci. Rep. 6:32980. 10.1038/srep32980 27622870 PMC5020685

[B53] Zhao Q. Guo Q. Liang X. Chen M. Zhou Y. Ding D.. (2015). Auditory verbal learning test is superior to rey-osterrieth complex figure memory for predicting mild cognitive impairment to Alzheimer's disease. Curr. Alzheimer Res. 12, 520–526. 10.2174/1567205012666150530202729 26027810

[B54] Zuo X. N. Ehmke R. Mennes M. Imperati D. Castellanos F. X. Sporns O.. (2012). Network centrality in the human functional connectome. Cereb. Cortex 22, 1862–1875. 10.1093/cercor/bhr269 21968567

